# Electrophysiological Evidence of Anticipatory Cognitive Control in the Stroop Task

**DOI:** 10.3390/brainsci11060783

**Published:** 2021-06-13

**Authors:** Valentina Bianco, Marika Berchicci, Elena Mussini, Rinaldo Livio Perri, Federico Quinzi, Francesco Di Russo

**Affiliations:** 1 Department of Languages and Literatures, Communication, Education and Society, University of Udine, 33100 Udine, Italy; 2 IRCCS Fondazione Santa Lucia, 00179 Rome, Italy; francesco.dirusso@uniroma4.it; 3 Department of Movement, Human and Health Sciences, University of Rome “Foro Italico”, 00135 Rome, Italy; m.berchicci@gmail.com (M.B.); elena.mussini@outlook.com (E.M.); perri.rinaldo@gmail.com (R.L.P.); fquinzi@libero.it (F.Q.); 4 Department of Psychology, University “Niccolò Cusano”, 00166 Rome, Italy

**Keywords:** stroop, proactive control, human cognition, anticipation, ERP, brain recording

## Abstract

The Stroop task has been largely used to explore the ability to inhibit the automatic process of reading when reporting the ink color of incongruent color-words. Given the extensive literature regarding the processes involved in task performance, here we aimed at exploring the anticipatory brain activities during the Stroop task using the event-related potential (ERP) method. To accomplish this, eighteen participants performed two different blocks where neutral words were intermixed with congruent and incongruent words, respectively. Results revealed consistent pre-stimulus activity over the frontal, premotor and parietal brain areas. The premotor and the parietal activities were also modulated by the Stroop effect, being more enhanced in the incongruent than in the congruent blocks. Present findings add on the current literature pointing at an unexplored locus of anticipatory cognitive control during task preparation, thus offering a new way to investigate top-down preparatory processes of performance control in the Stroop task.

## 1. Introduction

Our cognitive system continuously processes a large amount of information, even decoding simultaneous perceptual features in limited timeframes. To avoid overloads, we can voluntarily orient our selective attention to the relevant target by inhibiting the irrelevant one. This dual control of selective attention (i.e., voluntary vs. automatic) is orchestrated by the executive functions, acting in a flexible and goal-directed manner and allowing us to counteract the constraints of automatic processes [1]. This flexible behavior depends on our ability to cope with distracting stimuli that can interfere with the original goal. Specifically, this depends on the voluntary inhibition of distractors that may affect the simultaneous processing of target stimuli. The most popular task of automatic response inhibition is the Stroop test [2], in which participants are required to inhibit automatic reading while reporting the ink color of the word-stimulus. When the word-content and the ink color are incongruent, the performance is typically worse compared to the congruent condition, which gives rise to the ‘Stroop effect’ [3]. Despite almost one hundred years of Stroop literature, the considerable interest maintained in this task derives from its utility as a diagnostic and research tool to probe executive functions in healthy populations and in neurological patients [4,5,6].

A crucial debate in Stroop literature concerns the locus of cognitive interference [7,8]. However, there is good agreement that there are at least three loci: stimulus processing, semantic level, and response selection stages [9,10]. A substantial contribution to this debate has been made by event-related potential (ERP) studies, taking advantage of the high temporal resolution [8,11,12,13,14,15,16,17,18,19]. In this technique, electroencephalogram (EEG) recorded during a task is time-locked to specific events like the sensory stimulus to obtain EEG segments that, after averaging procedures, allow us to obtain specific brain responses associated with the event. The time-locking allows the segmentation of the signal before and/or after the specific event, allowing the analysis of both the pre-stimulus and post-stimulus ERP waveforms. All the above-mentioned ERP studies focused on reactive processing, but, as for any cognitive function, preparatory (proactive) processing strongly contributes to behavior. Proactive cognitive control requires top-down processing, as attention, and inhibition, to better interact with upcoming events. Recent literature has proposed an attentional inhibitory control (AIC) model [20], which attempts to reconcile the contribution of visual attention and inhibitory control mechanisms for properly engaging braking processes in our brain when appropriate.

Bugg and Jachoby [21] described distinct cognitive control mechanisms underlying Stroop performance: one control mechanism appears to operate rapidly and reactively on a trial-by-trial basis, acting after stimulus presentation; a second appears to operate strategically and proactively at block level, thus acting prior to stimulus onset. Based on current ERP literature, this proposal is difficult to test, and the reasons might be twofold: (i) pre-stimulus preparatory activities have been largely neglected in this field, and (ii) most Stroop studies have used a random trial design in which congruent/incongruent conditions were not predictable before stimulus presentation. To the best of our knowledge, the only available evidence is provided by two investigations on motor-related potentials, showing that these were not affected by Stroop interference [16,22] and by a recent study focusing on the effect of hypnotic suggestions on the preparatory brain processes [23]. Therefore, further research is needed to deepen our knowledge of proactive cognitive control in the Stroop test. In this regard, various preparatory slow cortical potentials have been acknowledged in the context of ERP studies using different motor/cognitive tasks: the Bereitschaftspotential (BP, [24]) preceding voluntary movement; the contingent negative variation (CNV, [25]) indexing different aspects of cognitive processing (i.e., perceptual processing of the cue, temporal expectancy, action readiness) occurring between a cue and an imperative stimulus; the stimulus-preceding negativity (SPN, [26]), reflecting the wait for knowledge of results or affective stimuli. Focusing on the BP, this component has typically been obtained as movement-related potential for self-paced movements, thus necessarily referred to motor activity; however, in light of its slow development, several studies have demonstrated that this component can also be obtained in relation to stimuli in sensory-motor tasks (for a review, see [27]). More recently, the existence of pre-motor anticipatory activities has been integrated with pre-stimulus ERPs related to proactive inhibition (i.e., the prefrontal negativity or pN, [27,28]) and sensory-related modality-specific anticipation [29].

In this context, the go/no-go task has largely been used, considering the involvement of proactive control mechanisms required to properly ensure the correct interaction between response and inhibition processes in the motor system [27]. However, although the go/no-go task challenges the response inhibition [30], it might also share some mechanisms with the Stroop test in that both require analysis of the visual stimulus, selective and sustained attention, and response inhibition. In addition, there is also behavioral evidence that scores of the two tasks are not correlated, suggesting different aspects of selective attention and response inhibition during task performance [31].

Taken together, the ERP literature on anticipatory processes bring to light the urge to challenge the role of the pre-stimulus activities for the Stroop test performance. Indeed, this will contribute to a better understanding of the neural basis of the anticipatory brain activities occurring during tasks challenging motor behavior.

In conclusion, the present ERP study aims to investigate the contribution of pre-stimulus brain processing on the resolution of the Stroop effect by providing a fixed block paradigm in which stimulus condition is always predictable (i.e., participants are informed about the congruency at the beginning of each block). Specifically, we hypothesize that (i) the Stroop task will require substantial cognitive and motor preparatory activity indexed by the pN and the BP components as previously described in different decisional tasks. In addition, considering the bimanual nature of the present task, (ii) we expect to find further preparatory activity associated with visuo-motor coordination in the parietal cortex, as was found in previous studies on bimanual coordination [32,33,34,35]. Regarding the Stroop effect, (iii) we expect that the incongruent condition could require enhanced top-down processing in brain areas subtending cognitive control.

## 2. Methods

### 2.1. Participants

Eighteen right-handed adults (8F, 26.0 ± 6.8 years, range 19–39) took part in the experiment. We determined the sample size through the G*POWER software (Allgemeine Psychologie und Arbeitspsychologie, Heinrich-Heine-Universität Düsseldorf, Germany) [36] based on the results of a previous study investigating ERPs markers of physical activity during the Stroop test [37]. We estimated a medium effect size of f (U) = 0.77, set the significance level to α = 0.05, and the desired power (1 − β) at 0.80 (estimated sample size =17). The value of the effect size was derived from the partial eta squared associated with the effect of congruency of the considered study and then converted using the SPSS function provided in the software. Participants were recruited at the University of Rome “Foro Italico”, Italy and were all fluent in the Italian language. Inclusion criteria were the following: age range 18–40 years, normal or corrected-to-normal vision, absence of any neurological or psychological disorders, and fluency in the Italian language. After explanations of the procedures, written informed consent was obtained from all participants according to the Declaration of Helsinki after approval by the Santa Lucia Foundation Ethical Committee. All participants were naïve to the aims and hypothesis of the experiment. Only after ending the experimental session were participants informed of the experimental hypothesis.

### 2.2. Apparatus and Task Procedure

**Figure 1:**
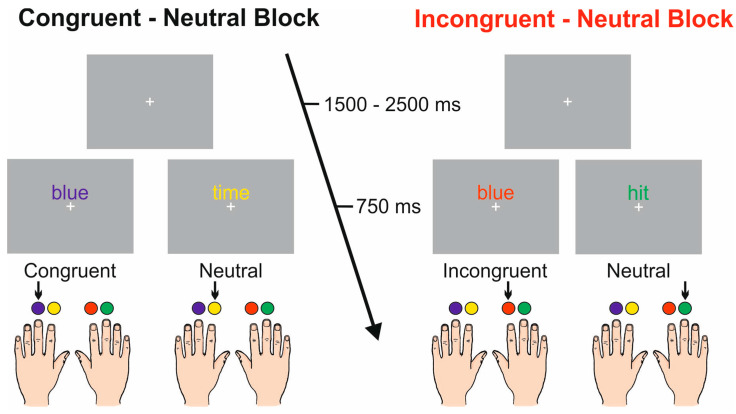
Representation of the experimental design of the two blocks.

Stimuli were presented via the Presentation Software (Neurobehavioral Systems, Inc. Berkeley, CA, USA). Figure 1 shows a sketch of the experimental design. An Italian version of the task was administered. Stimuli consisted of Italian written words in four possible ink colors, presented 0.5 cm above a white fixation cross in the center of a grey computer screen. The words were the Italian translations of “red”, “blue”, “yellow”, and “green” (“rosso”, “blu”, “giallo”, and “verde” in Italian) printed with a congruent ink color (e.g., word “red” printed in red) or with an incongruent ink color (e.g., word “red” printed in blue). Non-color words (English: “time”, “hit”, “rigid”, and “epoch”; Italian: “tempo”, “colpo”, “rigido”, and “epoca”) were also displayed using the same four ink colors. At the beginning of each block, participants were informed about the congruency of the upcoming block. The words subtended 1.0° of visual angle horizontally and 0.3° vertically, were presented individually, in lowercase, in Arial font and size 36, just above the fixation point, which subtended 0.15 × 0.15° of the visual angle.

In the present task, a four-choice manual version of the Stroop Test was used. Indeed, in contrast to the traditional Stroop Test requiring verbal responses, the manual task allows the analysis of motor behavior components associated with button pressing. Participants were seated in front of a screen placed 114 cm from the eyes with both hands positioned palm down on a push button board, so that the fingers could freely move on it. Responses consisted of pressing one of the four buttons corresponding to each of the four colors, which were mounted on the response box. The buttons were operated with the index and middle fingers of both hands. Participants were instructed to maintain their gaze on the fixation cross throughout the experiment and respond to stimuli as quickly and accurately as possible by pushing the colored button matching the ink color of the delivered words. Stimuli appeared for 750 ms and the inter-stimulus interval (ISI) was 1.5–2.5 s. The whole session consisted of two experimental blocks, each one presenting two stimulus categories: congruent-neutral and incongruent-neutral. Each block consisted of six runs, and each run consisted of 72 trials, for a total of 432 trials delivered for each block. Within each run, the couple of stimuli was randomly presented with a 0.5 probability, and the order of the runs was randomized among participants. The neutral words coupled with the congruent or incongruent color words were defined as neutral-C and neutral-I trials, respectively.

### 2.3. Behavioral Measures

Performance speed was assessed using the individual median response time (RT) for correct trials and the mean RT at group level. Accuracy was assessed using commission errors (CE) percentage. It must be noted that, in the present study, the main focus of the investigation is on the pre-stimulus EEG activity. Therefore, congruent and neutral trials, as well as incongruent and neutral trials were averaged separately for a direct comparison of ERPs across experimental blocks (please see next session). In this way, the RT of the congruent and incongruent blocks were compared through t-test for dependent samples.

### 2.4. EEG Recording and Analysis

All participants were individually tested in a sound attenuated, dimly lit room using an 80-channel EEG system (Brainamp™ amplifiers) with 64 active scalp electrodes (Acticap™) and software (Recorder 1.2 and Analyzer 2.2), all by BrainProducts GmbH (Munich, Germany). The scalp electrodes were mounted according to the 10–10 International System and initially referenced to the left mastoid (M1) and re-referenced to M1-M2 average. Horizontal and vertical electrooculograms (EOG) were monitored by bipolar recordings. The EEG was digitized at 250 Hz, amplified (bandpass of 0.01–60 Hz including a 50 Hz notch filter), and stored for offline averaging. The removal of eye movement artifacts was performed using the independent component analysis (ICA) ocular correction [38]. Data were high-pass (0.1 Hz) and low-pass (30 Hz) filtered and a semi-automatic artifact rejection was performed prior to signal averaging in order to discard epochs contaminated by signals exceeding the amplitude threshold of ±60 μV. Continuous EEG was segmented in epochs starting 1600 ms prior to the stimulus onset and lasting for 1900 ms and a baseline correction was applied in the interval −1600/−1400 ms prior to stimulus onset.

To limit multiple comparisons, we select the region of interest (ROI) and the time windows to include in the statistical analysis using the collapsed localizers method [39] and global field power (GFP). As reported by Skrandies [40] the measure of global field power (GFP) corresponds to the spatial standard deviation, and it quantifies the amount of activity at each time point in the field considering the data from all recording electrodes simultaneously, resulting in a reference-independent descriptor of the potential field. Following this method, the two blocks (congruent and incongruent) were averaged and the GFP was inspected with a t-test against zero, which was significantly different from zero starting from −1200 ms. Consequently, the mean amplitude of this interval (−1200/0 ms) was used for further analyses. In this interval, five main foci of negative activity were present: a pair of bilateral frontal foci labelled *prefrontal negativity* (pN); a medial centro-parietal focus associated with *the BP*; and a pair of bilateral parietal foci labelled as *posterior BP* (pBP). For this reason, the analysis was performed using the following ROIs constituted by electrode pools best representing the detected foci of activities: for the left pN the FC5-F5-F7 pool (left frontal) and for the right pN the FC6-F6-F8 pool (right frontal) were used. For the BP, the Cz-CPz-Pz (Medial Central) pool was used. For the left pBP the P3-P7-CP5 pool (left parietal) and for the right pBP the P4-P8-CP6 pool (right parietal) were used. Since congruent and neutral trials were unpredictable because they were equally and randomly presented in one block (labelled congruent block), and incongruent and neutral trials were equally and randomly presented in the other block (labelled incongruent block), pre-stimulus ERPs were obtained, averaging the two conditions in each block. For the pN and pBP, 2 × 2 ANOVAs were performed with block (congruent vs incongruent) and hemisphere (left vs right) as factors. For the BP, a t-test for dependent samples was performed with block as factor. For all statistical analyses, post-hoc comparisons, where appropriate, were carried out using the Bonferroni post-hoc test (dividing the *p*-value for the number of the used comparisons). The Cohen’s d and the partial eta squared (_p_η^2^) were used to measure the effect size of the significant effects for t-test and ANOVA, respectively. The overall alpha value was fixed at 0.05. Further, voltage and current source density (CSD) maps were obtained to better describe the scalp distribution of the studied components.

### 2.5. Correlation Analysis

Pearson’s correlations were performed between pre-stimulus ERPs amplitudes (i.e., BP, left and right pN, and pBP), and behavioral measures (i.e., the individual RT and the individual CE).

## 3. Results

### 3.1. Behavioral Results

The behavioral data of the two conditions are presented in Table 1. The t-test on RT showed a significant difference between the incongruent and congruent blocks, as expected (t_17_ = 6.388, *p* < 0.001, d = 1.205). Further, the t-test on accuracy rates showed a significant different between CEs of the congruent and incongruent blocks (t_17_ = 3.782, *p* < 0.001, d = 4.554).

### 3.2. Electrophysiological Results

**Figure 2:**
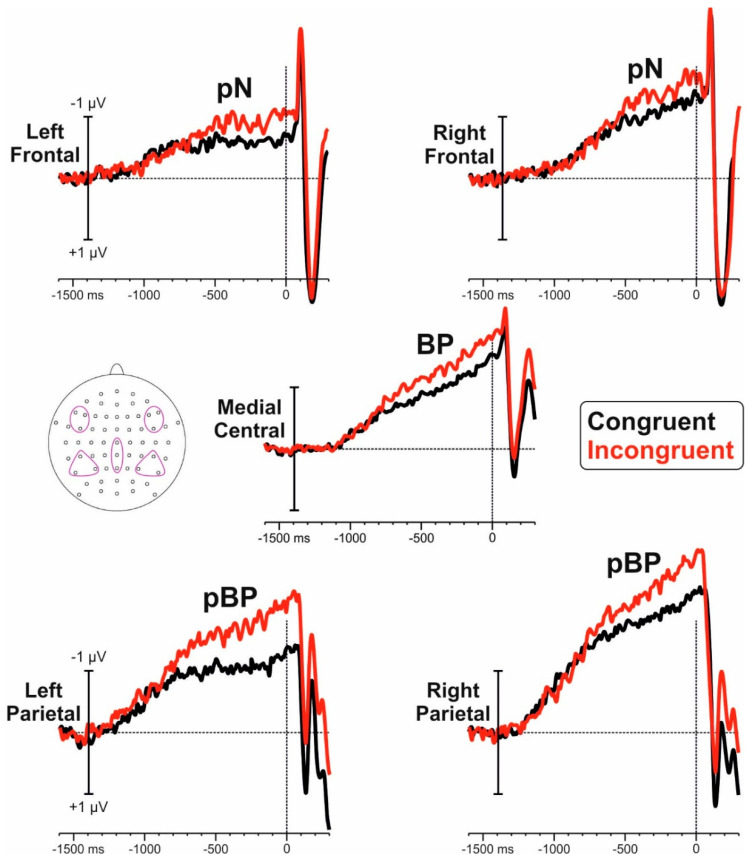
Pre-stimulus ERP waveforms from the five selected ROIs specified in the inset.

**Figure 3:**
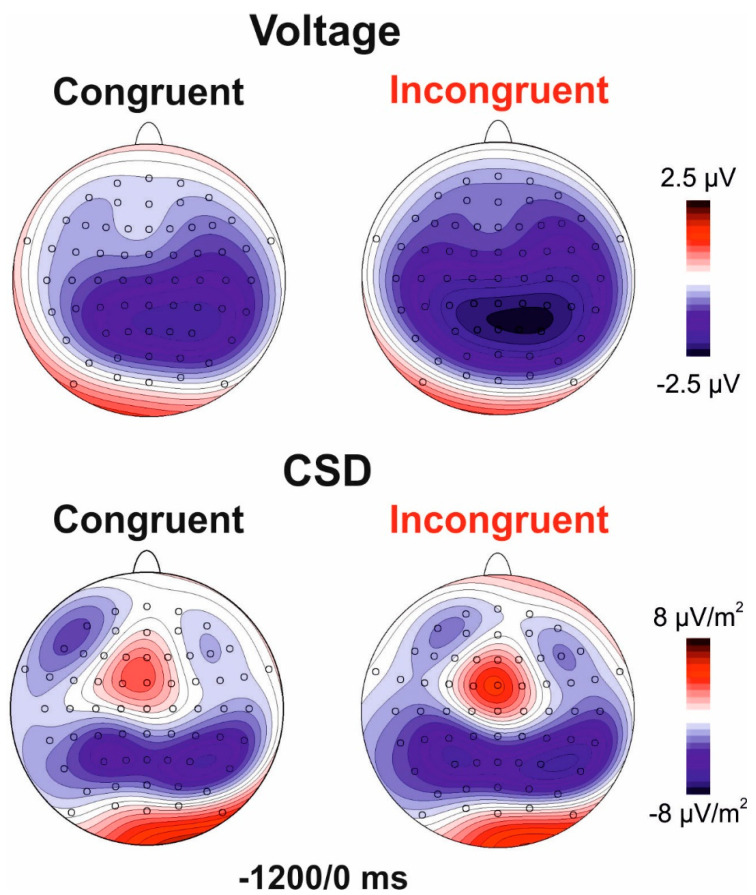
Pre-stimulus scalp topography. Top row: voltage distribution maps; bottom row: current source density (CSD) maps.

Figure 2 shows the pre-stimulus ERP for congruent and incongruent blocks. Figure 3 shows the voltage and CSD topographical distribution in the −1200/0 ms interval. Both blocks showed slow negative ramp-like activities in bilateral parietal regions starting at approximately −1200 ms and labelled as posterior BP (pBP). This activity was followed by the typical BP observed at medial centroparietal sites (onset −1100 ms) and by the prefrontal negativity (pN) at bilateral frontal sites initiating at about −1000 ms. The pN component is more clearly visible in the CSD maps.

#### 3.2.1. Posterior BP

Statistical analysis of the pBP showed a significant effect of hemisphere (F_1,17_ = 7.2 *p* = 0.015, _p_η^2^ = 0.297), block (F_1,17_ = 6.2, *p* = 0.023, _p_η^2^ = 0.266), and interaction (F_1,17_ = 7.9, *p* = 0.012, _p_η^2^ = 0.319); the pBP amplitude was larger for incongruent than congruent condition for both left (−1.37 µV vs −0.92 µV, *p* < 0.001) and right (−1.77 µV vs −1.54 µV, *p* = 0.003).

#### 3.2.2. BP

T-test for the BP was significant (t_17_= 2.34, *p* = 0.032, d = 0.255), with larger amplitudes for the incongruent (−1.68 µV) compared to the congruent block (−1.26 µV).

#### 3.2.3. pN

Statistical analysis of the pN did not show a significant effect of block (F_1,17_ = 2.5, *p* = 0.129), hemisphere (F_1,17_ = 1.3, *p* = 0.263), or interaction (F_1,17_ = 0.8, *p* = 0.393).

### 3.3. Correlation Results

Pearson’s correlation between the right pBP and RT for the incongruent condition was significant (*r* = 0.48, *p* < 0.05), indicating that the more negative the right pBP, the faster the RT.

## 4. Discussion

The novelty of the present study is the ERP investigation of the expectancy stage of processing in the Stroop task. The main aim is the identification of the anticipatory brain processing allowing the participants to prepare performance in experimental blocks characterized by the presence or absence of the Stroop conflict. Present data suggest that the brain preparation for the Stroop task involves a fronto-parietal network, and, crucially, the preparation for the incongruent condition involves larger anticipatory resources than the congruent one in premotor and parietal areas, but not in frontal areas.

Accordingly, a bilateral negative activity was also detected over the parietal areas and labeled posterior BP (pBP). This ERP was larger for the incongruent than congruent condition, as well as on the right than the left hemisphere. The pBP was distributed over lateral parietal areas and resembles the posterior BP described during the preparation of motor pantomimes [41] and grasping movements [33]. This component has been localized in the superior parietal lobe, and it was associated with planning of hand movements [33,34], including bimanual coordination [35,36] and stepping [42], but also the engagement of visuospatial attention in task processing [43]. The present task, requiring the coordination of two fingers per hand, may require substantial parietal preparation, which is also enhanced by the Stroop effect. Further, in line with a prominent view suggesting the parietal regions as a “command function” for motor plans [44,45] we might interpret the enhanced pBP in the incongruent condition as an index of increased effort to coordinate the motor effectors. This increased activity may be a consequence of the expectation of the upcoming conflict between the automatic and the incongruent responses. The larger activity in the right than in the left hemisphere may be due to the larger effort coordinating the non-dominant left hand [36]. Last, the presence of a correlation between the right pBP and RT in the incongruent condition also points to an increased involvement of the right rather than the left parietal cortex in determining Stroop task performance for more complex tasks.

Similar to the pBP component, the BP amplitude was increased in the incongruent block. To the best of our knowledge, this is the first study acknowledging the occurrence and the modulation of BP activity during Stroop task performance. One previous investigation studied the unfolding of the lateralized readiness potential (LRP, [46]), a measure of motor activation, but the authors mainly focused on this component as an index of incorrect response activation [47]. The BP has been largely interpreted as an index of motor readiness [48], with its source in supplementary motor areas (SMA, [27]). Longstanding literature described the BP as the brain correlate of proactive motor control during self-paced [49] and externally triggered motor-tasks [50] as the present visuo-motor response task. Considering that in these types of tasks the increased BP has been associated with discrimination complexity [51], we suggest that the enhanced BP amplitude in the incongruent block is due to the greater engagement of the inhibitory control network, of which the SMA is part [52].

The negative activity emerging at frontal sites (pN) is similar to that observed in previous studies in discriminative response tasks [27,28] and interpreted as the ERP correlate of proactive top-down attentional control [20,53], which is crucial for task accuracy [50]. In contrast with our hypothesis, this component was not sensitive to the different demands between the congruent and incongruent blocks, thus challenging the involvement of proactive top-down control; however, this component might also reflect the level of generic, sustained attention in the expectancy stage of processing required to prepare a cognitively demanding task, as also previously demonstrated in a previous study [54]. Further, given that the observed activities emerge on more lateral sites (i.e., dorsolateral) than previously reported (i.e., mainly medial prefrontal), an alternative account deserves further consideration. Indeed, these could reflect activity of the dorsolateral prefrontal cortex (dlPFC), an area consistently involved during the execution of several tasks challenging sustained and selective attention [55,56,57]. Crucially, a neurostimulation study revealed that the dorsolateral portion of the PFC was the only one that, when stimulated, produced a detectable alteration in performance in the Stroop test [58]. Also, the dlPFC shares many functional properties with the posterior parietal cortex, and these areas are co-activated in a range of cognitive operations requiring visuospatial attention [59]. Therefore, in the present Stroop task, the observation of dorsolateral (the pN) and parietal (pBP) activities during the preparation stage of processing point at an early involvement of selective attention mechanisms, which are presumably activated in advance in order to ensure the proper resolution of the word-color interferences prompted by this specific task. This proposed view is further in line with the Cascade-of-Control model [60,61], which claims that posterior portions of the lateral PFC might enact the attentional set in the Stroop test, upregulating color processing and/or downregulating word processing even prior to stimulus onset, thus implementing proactive control. Accordingly, the lateral PFC would interact with posterior brain regions to ensure the selection of the relevant information (i.e., color) at the expense of the irrelevant one (i.e., word). Lastly, the existence of pre-stimulus processes occurring during the Stroop test has been finely demonstrated by Kalanthroff and co-workers [62]. Indeed, they showed that a concurrent working memory task deeply diminished proactive control mechanisms necessary to focus on the relevant dimension of the color word.

In line with longstanding literature on the Stroop test, response times were slower for the incongruent compared to the congruent block. Namely, word meaning influenced responses to the ink color even though it was irrelevant for task performance [7].

## 5. Conclusion and Future Perspectives

Overall, the present results showed that the Stroop effect on cognitive functions is not limited to the reactive stage of processing, but it also involves proactive pre-stimulus activity [20]. In particular, the parietal and premotor brain areas play a key role in the proper preparation for cognitive conflict resolution. This evidence unveils a novel type of anticipatory neural set for the cognitive interference resolution, and calls for future investigations aiming at further exploring endogenous, proactive brain activity in both normal and neurological populations. Indeed, a plethora of studies provide encouraging findings in considering that the modulation of slow-wave pre-stimulus ERP activities (including the components considered here) might predict motor and cognitive performance during discrimination tasks (e.g., [27,63,64,65]). Given that in the pre-stimulus stage of processing, crucial hints of future action performance occur, a better understanding of the underlying proactive brain activities in normal individuals could yield findings of critical diagnostic importance, especially for certain movement disorders [66]. Last, we have recently found that the cognitive load may anticipate the onset and enhance the amplitude of the anticipatory ERP components [51], and that this might also partially explain the observed differences between blocks. Therefore, upcoming studies are encouraged to consider the possible modulatory effects of stress, anxiety, and cognitive load on proactive control mechanisms in similar tasks prompting fast and accurate performance.

## Figures and Tables

**Table 1:** Response time (RT), percentage of commission errors (CE), and relative standard error (SE).

	RT ± SE	CE ± SE
Incongruent	549 ± 10	7.2 ± 1.2
Congruent	505 ± 8	5.1 ± 0.9

## Data Availability

Data are available from the corresponding author upon request.
